# Clustered Protocadherins Emerge as Novel Susceptibility Loci for Mental Disorders

**DOI:** 10.3389/fnins.2020.587819

**Published:** 2020-11-12

**Authors:** Zhilian Jia, Qiang Wu

**Affiliations:** Center for Comparative Biomedicine, MOE Key Laboratory of Systems Biomedicine, State Key Laboratory of Oncogenes and Related Genes, School of Life Sciences and Biotechnology, Institute of Systems Biomedicine, Shanghai Jiao Tong University, Shanghai, China

**Keywords:** axonal tiling and myelination, neuronal self-recognition and dendrite self-avoidance, cell adhesion, neuropsychiatric diseases, neuronal connectivity, gene dysregulation, 3D genome architecture, clustered protocadherins

## Abstract

The clustered protocadherins (cPcdhs) are a subfamily of type I single-pass transmembrane cell adhesion molecules predominantly expressed in the brain. Their stochastic and combinatorial expression patterns encode highly diverse neural identity codes which are central for neuronal self-avoidance and non-self discrimination in brain circuit formation. In this review, we first briefly outline mechanisms for generating a tremendous diversity of cPcdh cell-surface assemblies. We then summarize the biological functions of cPcdhs in a wide variety of neurodevelopmental processes, such as neuronal migration and survival, dendritic arborization and self-avoidance, axonal tiling and even spacing, and synaptogenesis. We focus on genetic, epigenetic, and 3D genomic dysregulations of *cPcdhs* that are associated with various neuropsychiatric and neurodevelopmental diseases. A deeper understanding of regulatory mechanisms and physiological functions of *cPcdhs* should provide significant insights into the pathogenesis of mental disorders and facilitate development of novel diagnostic and therapeutic strategies.

## Introduction

The assembly of complex neural circuits, which are essential for accurate processing of sensory and cognitive information, requires individual neurons to discriminate self from non-self. This neuronal discrimination is thought to be accomplished by cell identity codes – neural recognition addresses determined by specific cell-surface receptors (Zipursky and Grueber, 2013). Given that there are billions of neurons each forming up to twenty thousand synapses in the human brain (Pakkenberg and Gundersen, 1997; DeFelipe et al., 1999), it’s fascinating how neurons use limited number of genes to generate such seemingly unlimited diversities. Failure in generating diversified neuronal codes causes deficits in neural circuit formation and may be related to pathogenesis of brain disorders.

Vertebrates adopt several mechanisms to achieve molecular diversity in different systems. For example, in the immune system, individual B and T cells express distinct repertoires of immunoglobulin (*Ig*) and T-cell receptor (*TCR*) genes through stochastic V(D)J rearrangements in the *Ig* and *TCR* gene clusters, respectively (Tonegawa, 1983; Wu et al., 2020). In the olfactory system, each olfactory sensory neuron expresses a single olfactory receptor (*OR*) out of more than a thousand *OR* genes. In this case, interchromosomal interactions bring 63 *OR* enhancers (Greek islands) together to activate one and only one *OR* gene in each neuron (Monahan et al., 2019). In the nervous system, more than 1,000 distinct isoforms of neurexins are generated by alternative splicing (Ullrich et al., 1995). In addition, the clustered protocadherins (cPcdhs), the largest subfamily of cadherin superfamily proteins, endow each individual neuron with a unique identity code (Wu and Maniatis, 1999). The molecular diversity of cPcdhs is generated by stochastic promoter choice and combinatorial expression regulated by the genome architectural protein CTCF (CCCTC-binding factor) (Golan-Mashiach et al., 2012; Guo et al., 2012, 2015; Monahan et al., 2012; Jia et al., 2020).

The expressed Pcdh isoforms are proposed to form repertoires of dimers on the cell membrane through promiscuous *cis*-interactions, as observed in crystal structures (Rubinstein et al., 2015; Goodman et al., 2016b, 2017; Brasch et al., 2019). When contacting with opposed neurites expressing the same combinations, the *cis*-dimer repertoires are thought to assemble into a large zipper lattice through stringent homophilic *trans*-interactions (Schreiner and Weiner, 2010; Thu et al., 2014; Brasch et al., 2019). Thus, the cPcdhs mediate specific cell-cell adhesion through these *trans*-interactions and are strong candidates for cell-surface neuronal identity codes (Wu and Maniatis, 1999; Thu et al., 2014; Brasch et al., 2019). In addition to *cPcdhs*, non-clustered Pcdhs also mediate cell adhesion through homophilic *trans*-interactions (Honig and Shapiro, 2020). Clustered and non-clustered Pcdhs may have cooperative functions in brain development.

Consistent with their prominent expression in the nervous system, cPcdh proteins function in many processes during brain development and are thought to be important for the construction of neuronal connectivity leading to the adult brain (Wang et al., 2002b; Weiner et al., 2005; Garrett and Weiner, 2009; Lefebvre et al., 2012; Katori et al., 2017; Mountoufaris et al., 2017; Fan et al., 2018). Emerging evidence suggests that genomic variants, epigenetic alterations, and 3D genome architectural dysregulation of the *cPcdhs* are associated with a wide variety of brain disorders in both animal models and human patients. In this review, we specifically focus on recent progress of the roles of cPcdhs in brain development and their dysregulation in brain disorders. We propose that epigenetic dysregulation of cPcdhs underlies many mental disorders. We refer interested readers to other recent reviews describing various aspects of cPcdhs (Flaherty and Maniatis, 2020; Honig and Shapiro, 2020; Pancho et al., 2020; Sanes and Zipursky, 2020; Wu and Jia, 2020).

## Molecular Mechanisms for Generating Diversified Neural Identity Codes

### Promoter Choice of cPcdhs in the Cell Nucleus

**FIGURE 1:**
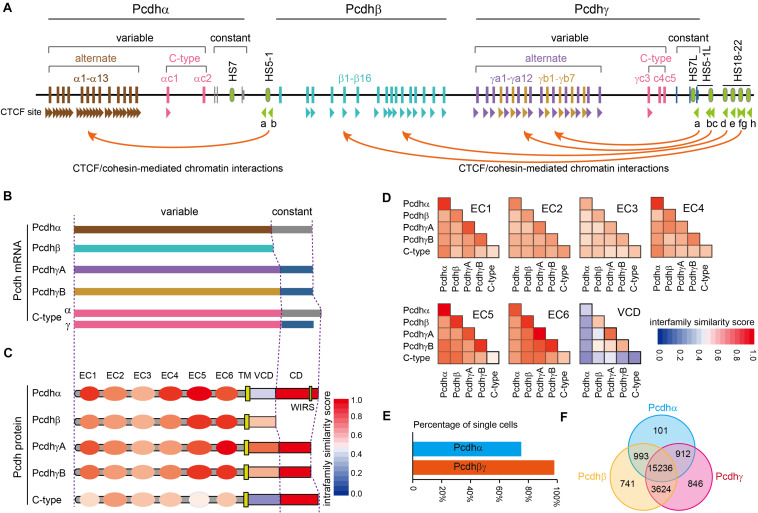
Characteristics of the human clustered *Pcdhs*. **(A)** The unique genomic organization of the three closely-linked human *Pcdh* gene clusters. The *Pcdh*α and *Pcdh*γ clusters are organized into variable and constant regions while the *Pcdh*β cluster contains only variable region with no constant region. The *Pcdh*α cluster contains 15 variable exons (13 alternate exons of *α1*-*α13* and 2 C-type exons of *αc1* and *αc2*) each independently spliced to a single set of three downstream constant exons. Each *Pcdh*α variable exon is associated with its own promoter. Each *Pcdh*α alternate promoter carries two forward CTCF sites, *Pcdhαc1* carries one forward CTCF site, and *Pcdhαc2* carries no CTCF site. These forward CTCF sites function as topological insulators and form directional chromatin looping with the two reverse CTCF sites of the downstream HS5-1 enhancer. The *Pcdh*β cluster contains 16 variable exons of β*1*–β*16* each encoding a single Pcdhβ protein. Each *Pcdh*β variable exon is associated with its own promoter. Each *Pcdh*β promoter carries a forward CTCF site except β*1*. The *Pcdh*γ cluster contains 22 variable exons (19 alternate exons of 12 A-type: *γa1*–*γa12*; 7 B-type: *γb1*–*γb7*; 3 C-type exons: *γc3*–*γc5*) each independently spliced to a single set of three downstream constant exons. Each *Pcdh*γ variable exon is associated with a promoter. Each *Pcdh*γ promoter carries a forward CTCF site except *γc4* and *γc5*. These forward *Pcdh*β and *Pcdh*γ CTCF sites function as topological insulators and form directional chromatin looping with a tandem array of reverse CTCF sites of the downstream super-enhancer. Enhancer and super-enhancer are marked with green ovals. The location of CTCF sites and their orientation are shown as arrowheads. **(B)**. Diverse *cPcdh* mRNAs, which can be divided into five groups, are generated by a combination of stochastic promoter activation and alternative splicing. **(C)**. Shown is the domain organization of the encoded cPcdh proteins. Each variable exon of the *Pcdh*α, *Pcdh*β, and *Pcdh*γ clusters encodes an extracellular domain with a signal peptide and 6 ectodomains (ECs), a transmembrane domain (TM), and a variable cytoplasmic domain (VCD). The constant exons encode an intracellular common domain (CD) shared by all members of the *Pcdh*α or *Pcdh*γ cluster. The color in each domain represents the amino-acid sequence similarity score between isoforms of the same group. WIRS, Wiskott-Aldrich syndrome family verprolin homologous protein (WAVE) interacting receptor sequence. **(D)** The amino-acid sequence similarity of each domain between different groups. Note that the EC2/EC3 are the most diversified ectodomains and EC5/EC6 are the most conserved ectodomains among all six ectodomains. VCDs are the least conserved. **(E)** Percentage of cortical cells expressing *Pcdh*α or *Pcdh*βγ. The *Pcdh*α isoforms are expressed in 74.5% of while the *Pcdh*βγ isoforms are expressed in nearly all of cortical cells. **(F)** Venn diagram of populations of single cells expressing *Pcdh*αβγ.

The unique genome organization of *cPcdh* genes has the potential to generate unparalleled diversities for neuron identity codes. The *cPcdh* locus contains three closely-linked gene clusters (*Pcdh*α, *Pcdh*β, and *Pcdh*γ) encompassing nearly one million base pairs at the human chromosome 5q31 region (Figure 1A) (Wu and Maniatis, 1999). Both the *Pcdh*α and *Pcdh*γ clusters contain tandem arrays of large variable exons, each of which is transcribed from its own promoter and then spliced to the common set of three downstream constant exons within the respective cluster (Figure 1A) (Wu and Maniatis, 1999; Tasic et al., 2002; Wang et al., 2002a). The *Pcdh*β cluster has no constant region and thus contains only variable exons (Figure 1A) (Wu and Maniatis, 1999; Wu et al., 2001). Each *Pcdh*α, β, and γ variable exon encodes an extracellular domain with 6 ectodomains (ECs), a transmembrane domain (TM), and a juxtamembrane variable cytoplasmic domain (VCD). The three constant exons encode a common membrane-distal cytoplasmic domain (αCD and γCD) (Figures 1B,C) (Wu and Maniatis, 1999). Based on phylogenetic analyses, the 53 human *cPcdh* genes are divided into five groups: *Pcdh*α (*α1* – *α13*), *Pcdh*β (β*1* – β*16*), *PcdhγA* (*γa1 – a12*), *PcdhγB* (*γb1* – *γb7*), and C-type (*αc1*, *αc2*, *γc3*, *γc4*, *γc5*) (Wu and Maniatis, 1999; Wu et al., 2001). Interestingly, different species, even between the closest relatives such as human and chimpanzee, do not have exact the same number of *cPcdh* genes, suggesting that their absolute numbers are not important once reached a certain threshold (Wu, 2005).

The three *Pcdh* clusters contain tandem arrays of CTCF binding sites (CBS) in both gene promoters and enhancers (Figure 1A) (Golan-Mashiach et al., 2012; Guo et al., 2012; Monahan et al., 2012). CTCF/cohesin-mediated long-range chromatin interactions between these CBS elements determine the stochastic and combinatorial expression of diverse cPcdh repertoires on the cell surface (Wu and Jia, 2020). Differential expression of *cPcdh* genes in individual neurons is regulated by epigenetic modifications, such as DNA methylation and H3K9me3 histone modification in the promoter region (Guo et al., 2012; Toyoda et al., 2014; Chen et al., 2015; Jiang et al., 2017; Wada et al., 2018). In particular, DNA methylation precludes the binding of CTCF proteins (Wu et al., 2001; Guo et al., 2012; Yin et al., 2017). Without CTCF binding, *cPcdh* promoters cannot form long-range chromatin contacts with remote enhancers and thus cannot be activated (Guo et al., 2012; Canzio et al., 2019). It is this higher-order chromatin architecture that determines the *Pcdh* promoter choice in the cell nucleus (Wu and Jia, 2020).

### Cell-Surface Delivery of cPcdhs Through Cytoplasm

Significant progress has been made on cell-surface delivery mechanisms of cPcdhs. In particular, Pcdhγ proteins are initially detected in the cytoplasm as a mobile pool and their cell-surface delivery corresponds to the maturation of synapses (Fernandez-Monreal et al., 2009, 2010; LaMassa et al., 2020). For example, Pcdhγa3 and Pcdhγb2 form juxtanuclear membrane tubules in the cytoplasm when overexpressed (Hanson et al., 2010). In contrast, Pcdhγc4 accumulates as continuous sheets but does not form tubules (Hanson et al., 2010). Intracellular trafficking and tubulation of Pcdhγa3 are determined by a 26-amino-acid VCD motif (O’Leary et al., 2011). Consistently, despite that the VCD domain is the most diverse region of cPcdhs (Figures 1C,D), there are several highly conserved residues within the VCD motif (Wu and Maniatis, 1999; Shonubi et al., 2015).

Pcdhαc2 and isoforms of the Pcdh β and γ families (except γc4) can be delivered to the cell surface by themselves; by contrast, surface localization of Pcdhα (except αc2) and Pcdhγc4 requires the co-expression with other isoforms of Pcdh β or γ, known as carrier isoforms (Murata et al., 2004; Bonn et al., 2007; Thu et al., 2014; Goodman et al., 2016b). The mechanism by which carrier isoforms facilitate the delivery of members of Pcdhα and Pcdhγc4 to the cell surface is not known but may require *cis*-dimerization on the ER membrane between cPcdh isoforms. This hypothesis is consistent with evidence of *cis*-dimerization or multimerization between cPcdhs in transfection experiments (Schalm et al., 2010; Schreiner and Weiner, 2010; Biswas et al., 2012; Thu et al., 2014; Rubinstein et al., 2015). Finally, analyses of single-cell RNA-seq data of 23,178 cortical cells (Tasic et al., 2018) revealed that almost every cell (97.0%) expresses isoforms of *Pcdh*β or γ but only 74.5% cells express members of *Pcdh*α (Figure 1E). Moreover, every *Pcdh*α-expressing cell (99.4%) co-expresses isoforms of either *Pcdh*β or γ, while only 76.7% of *Pcdh*β- or γ-expressing cells co-express members of *Pcdh*α, consistent with the requirement of Pcdh β or γ for the cell-surface delivery of Pcdhα (Figure 1F).

### Self-Recognition and Non-self Discrimination on the Cell Surface

**FIGURE 2:**
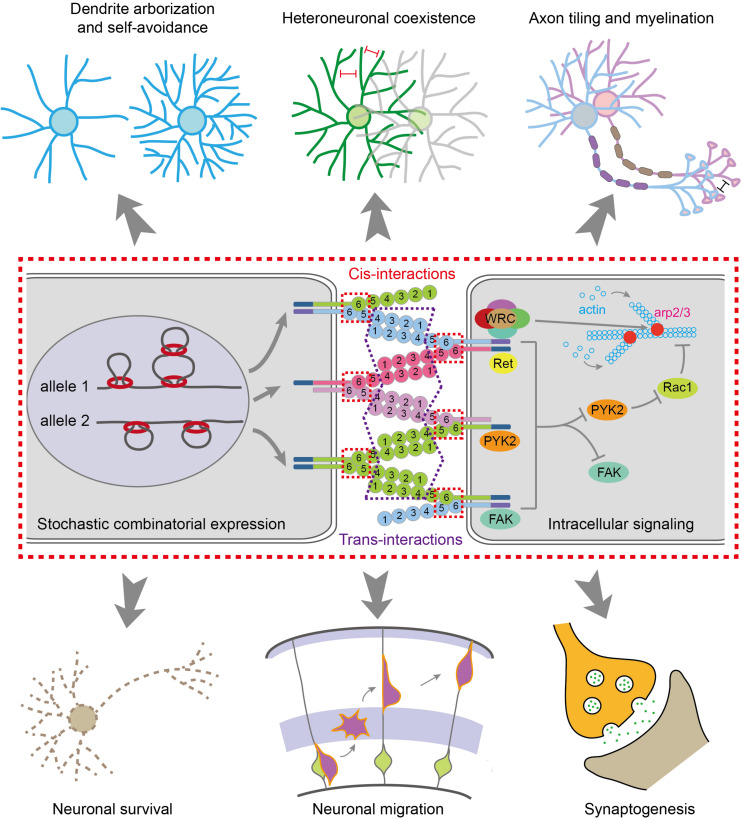
Molecular mechanisms for generating diverse neural identity codes. The stochastic and combinatorial expression of *cPcdhs* by CTCF/cohesin-mediated chromatin interactions generates vast repertoires of cell surface proteins. Most *cPcdh* genes are expressed monoallelically. The cPcdh proteins form *cis*-dimers and engage in specific homophilic *trans*-interactions. Through cytoplasmic common effectors of WRC, Pyk2, FAK, Ret and Rac1, *cPcdhs* regulate actin dynamics and cytoskeletal remodeling, eventually leading to various cellular functions, such as dendritic arborization and self avoidance, heteroneuronal neurite coexistence, axon tiling and myelination, neuronal survival and migration, and synaptogenesis.

The *cis*-dimers of cPcdhs can engage in highly stringent homophilic *trans*-interactions to generate cell-recognition specificities (Thu et al., 2014). The *trans*-interactions of cPcdhs are mediated through EC1–EC4 domains, especially the most-divergent EC2 and EC3 domains, in an anti-parallel manner (Figures 1C,D, 2) (Wu, 2005; Schreiner and Weiner, 2010; Nicoludis et al., 2015, 2016, 2019; Rubinstein et al., 2015; Goodman et al., 2016a; Brasch et al., 2019). Remarkably, liposome-tethered ectodomains spontaneously assemble into a zipper-like lattice structure (Brasch et al., 2019). Thus, when neurites bearing the same cPcdh isoforms contact with each other, the cPcdhs are thought to form a zipper lattice through homophilic *trans*-interactions and trigger an intracellular signaling pathway that eventually leads to adhesion or repulsion (e.g., self-avoidance) through cytoskeletal remodeling (Molumby et al., 2016; Fan et al., 2018; Brasch et al., 2019).

Isoforms of Pcdhα and Pcdhγ could be cleaved by the metalloproteinase ADAM10 and γ-secretase to release the intracellular domain which translocates into the nucleus to regulate gene expression (Haas et al., 2005; Hambsch et al., 2005; Reiss et al., 2006; Bonn et al., 2007; Buchanan et al., 2010). Thus, diverse extracellular signals can converge on the same intracellular signaling pathway through the common CD shared by members of *Pcdh*α or *Pcdh*γ (Wu and Maniatis, 1999; Wu et al., 2001; Schalm et al., 2010; Fan et al., 2018) (reviewed in Wu and Jia, 2020). In addition, the intracellular domains of Pcdhα and Pcdhγ can bind to several kinases including FAK (focal adhesion kinase), Pyk2 (proline-rich tyrosine kinase 2), and Ret (receptor tyrosine kinase rearranged during transformation) (Figure 2) (Chen et al., 2009; Schalm et al., 2010; Garrett et al., 2012; Suo et al., 2012; Keeler et al., 2015). Finally, Pyk2 inhibits the activities of Rac1, which regulates lamellipodial dynamics and actin cytoskeletal remodeling (Figure 2) (Suo et al., 2012; Fan et al., 2018).

## Biological Functions of Clustered Pcdhs

### Homophilic Adhesion-Induced Repulsion Is Required for Dendrite Self-Avoidance

**FIGURE 3:**
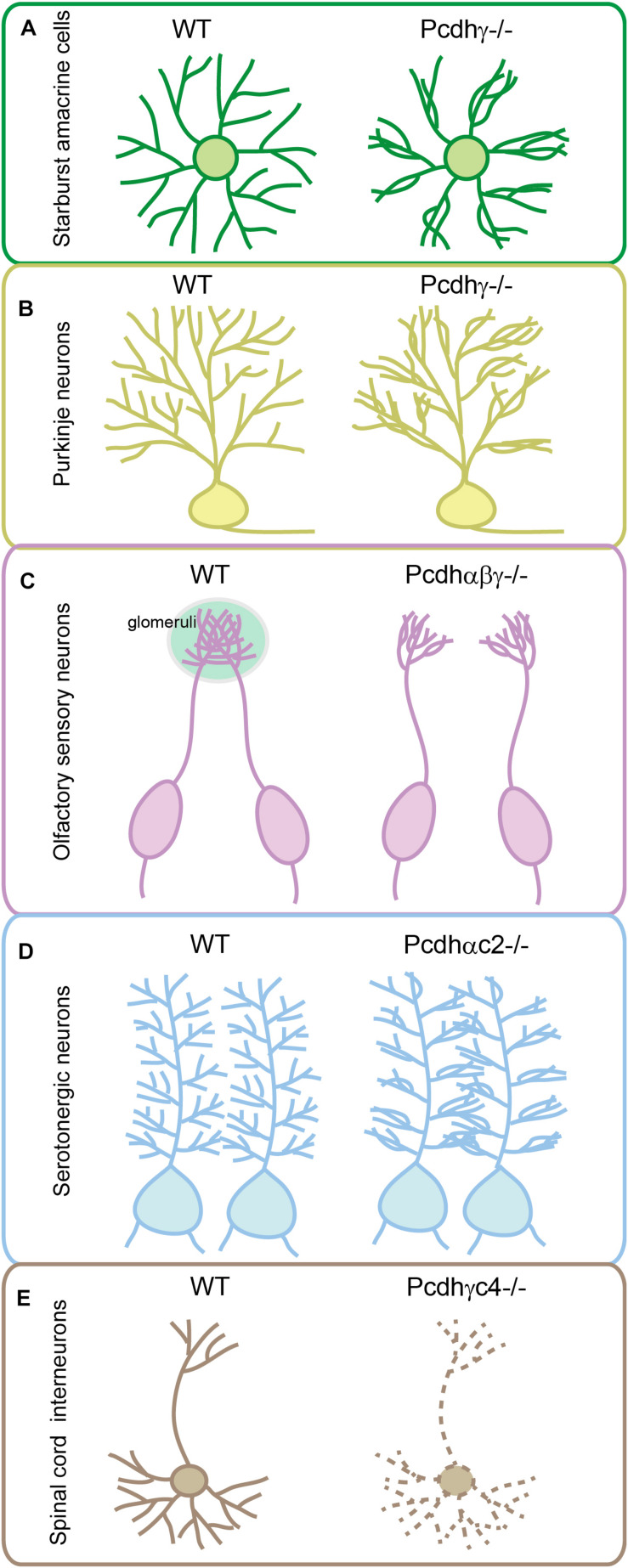
Genetic evidence for cPcdh functions in neuronal wiring. Pcdhγ has critical function in sister dendrite self-avoidance of the retinal starburst amacrine cells **(A)** and cerebellar Purkinje neurons **(B)**. Tri-cluster Pcdhs are required for olfactory sensory neurons to form glomeruli in the olfactory bulb **(C)**. Pcdhαc2 is essential for the even spacing and tiling of serotonergic axons **(D)**. Pcdhγc4 is the sole necessary and sufficient isoform for interneuron survival in the spinal cord **(E)**.

During development, sister neurites avoid each other in innervating the receptive field, a phenomenon known as self-avoidance. Neurite self-avoidance requires neurons to discriminate self from non-self (Grueber and Sagasti, 2010; Zipursky and Grueber, 2013). In *Drosophila*, self-avoidance is mediated through diversified *Dscam1* (Down syndrome cell adhesion molecule 1) generated by alternative splicing (Zipursky and Grueber, 2013). In vertebrates, it is thought to be achieved by diverse cPcdhs as supported by a growing body of evidence. In particular, knockout of *Pcdh*γ causes collapses of dendrites, a defect of self-avoidance, of the same starburst amacrine cells (Figure 3A) (Lefebvre et al., 2012; Kostadinov and Sanes, 2015). When the entire *Pcdh*γ cluster is replaced with a single *Pcdh*γ isoform, self-avoidance defects are rescued. However, these cells recognize heteroneuronal dendrites as ‘self’ since neighboring starburst cells also express the same cPcdh isoform (Lefebvre et al., 2012). Similar to starburst cells, dendritic self-avoidance defects were also observed in cerebellar Purkinje cells in the absence of *Pcdh*γ (Figure 3B) (Lefebvre et al., 2012; Toyoda et al., 2014). Subsequent studies demonstrated that *Pcdh*α and *Pcdh*γ function together to mediate dendritic self-avoidance of starburst and Purkinje cells (Ing-Esteves et al., 2018).

### Isoform Diversity Is Required for Neurite Coexistence

In addition to a critical role in dendritic self-avoidance, cPcdh diversity plays a crucial role in dendrite coexistence (Lefebvre et al., 2012). Specifically, reducing the diversity of Pcdhγ disrupts dendrite coexistence in retinal starburst amacrine cells (Lefebvre et al., 2012). The cPcdh proteins also function in patterning of axon terminals. For example, olfactory sensory neurons (OSNs) expressing identical OR genes project their axons to the same location in the olfactory bulb and converge to form a glomerulus (Mombaerts et al., 1996). Mechanistically, alternate cPcdh isoforms from all three *Pcdh* clusters are stochastically expressed in single OSNs, ensuring that axons from different OSNs do not share the same profile and thus can coexist in the same glomeruli of the olfactory bulb (Mountoufaris et al., 2017). Genetic deletion of the entire *Pcdh*α cluster in mice results in abnormal sorting of OSN axons leading to the formation of ectopic small glomeruli (Hasegawa et al., 2008; Mountoufaris et al., 2017). Deletion of *Pcdh*β or *Pcdh*γ also affects OSN wiring (Hasegawa et al., 2016; Mountoufaris et al., 2017). Finally, deletion of all three *Pcdh* clusters in mice causes the most severe phenotype with axons from the same OR-expressing OSNs branching profusely, reminiscent of the axonal self-avoidance defects (Figure 3C) (Mountoufaris et al., 2017).

Gain-of-function experiments provide additional insights into the role of cPcdhs in OSN wiring. Overexpression of the same three cPcdh isoforms (one isoform from each cluster) in different OSNs, which overrides the endogenous cPcdh diversity and makes every OSN express the same set of cPcdh isoforms, results in their axons repelling each other and projecting diffusely (Mountoufaris et al., 2017). These axons cannot converge in the same location of the olfactory bulb to form proper glomeruli. The severity of convergence defects correlates with numbers of neurons overexpressing *cPcdhs* (Mountoufaris et al., 2017).

### Pcdhαc2 Regulates Axonal Tiling of Serotonergic Neurons

Serotonin is a critical neurotransmitter that regulates diverse higher-order brain functions including mood, cognition, reward, learning and memory. Although there are only 300,000 serotonergic neurons in the human brain, serotonergic axon terminals innervate the entire brain with even spacing. This tilling pattern is required for the maintenance of constant serotonin levels since serotonin molecules can only function over a short distance from their releasing sites. Genetic evidence suggests that *Pcdh*α participates in the even spacing of serotonergic axons in mice (Chen et al., 2017; Katori et al., 2017). Specifically, deletion of the *Pcdh*α cluster, but not double deletion of the *Pcdh*β and γ clusters, causes serotonergic axons to form clumps throughout the brain and results in insufficient terminal arborization in mice (Chen et al., 2017; Katori et al., 2017). In addition, labeling individual serotonergic neurons with brainbow technologies revealed that serotonergic axons from different neurons have tiling and self-avoidance defects upon constitutive *Pcdh*α deletion (Chen et al., 2017). Finally, these *Pcdh*α-deleted mice displayed depression-like behaviors, including increased immobility time in tail-suspension, forced-swimming, and contextual fear conditioning tests (Chen et al., 2017).

Conditional knockout of the entire *Pcdh*α cluster in serotonergic neurons or their target fields revealed cell-autonomous functions of *Pcdh*α in serotonergic neurons (Chen et al., 2017; Katori et al., 2017). However, deletion of *α1-α12* of the *Pcdh*α cluster results in no obvious defect in serotonergic wiring, suggesting an important role of C-type *Pcdh*α genes in serotonin circuit formation. Consistently, deletion of both *Pcdhαc1* and *Pcdhαc2*, or deletion of *Pcdhαc2* alone, causes the clumping of serotonergic axons similar to deletion of the entire *Pcdh*α cluster (Chen et al., 2017; Katori et al., 2017). In addition, *Pcdhαc2* is predominately expressed in serotonergic neurons (Chen et al., 2017; Katori et al., 2017). Thus, among all members of the *Pcdh*α cluster, only *Pcdhαc2* has been shown to be required for serotonergic axon wiring (Figure 3D) (Chen et al., 2017; Katori et al., 2017). This is a striking example that a neuronal cell type uses a single cPcdh isoform to achieve axonal tiling or even spacing in all of its projecting fields. Therefore, in contrast to the diversity generated by multi-cPcdh isoforms for cell–cell recognition of starburst cells, Purkinje cells, and OSNs, the single-isoform-mediated repulsion is a remarkable strategy adopted by serotonergic axons to tile brain regions with even spacing. However, since Pcdhγ c4 and c5 are also expressed but at lower levels in serotonergic neurons (Chen et al., 2017), it is difficult to rule out their partially redundant contribution in patterning the serotonergic axons. Combinatorial C-type gene deletion and rescue experiments are needed to fully address this important question.

### Pcdhγc4 Is Required for Interneuron Survival

Experiments of *in situ* hybridization and immunohistochemistry showed that *Pcdhγc4* is expressed in many brain regions as well as in spinal cord (Zou et al., 2007; Miralles et al., 2020). Detailed analyses of constitutive or conditional mutant mice lacking all *Pcdh*γ genes revealed the increased apoptosis of interneurons in the spinal cord, retina, cerebral and cerebellar cortex (Wang et al., 2002b; Lefebvre et al., 2008; Prasad et al., 2008; Chen et al., 2012; Hasegawa et al., 2016; Carriere et al., 2020; Mancia Leon et al., 2020). For example, in retina, *Pcdh*γ deletion results in interneuron apoptosis but does not affect survival of neighboring WT interneurons, suggesting a cell-autonomous effect (Lefebvre et al., 2008). In the spinal cord, however, the survival of *Pcdh*γ-deficient interneurons can be enhanced if they are surrounded by neurons expressing *Pcdh*γ (Prasad et al., 2008). This non-cell autonomous effect on survival was also observed for cortical interneurons (Mancia Leon et al., 2020). Taken together, these studies suggest that the effect of Pcdhγ on interneuron survival is cell- or tissue-type dependent.

Knockout of the three C-type isoforms (*Pcdhγc3*-*c5*) of the *Pcdh*γ cluster results in neuronal apoptosis that is indistinguishable from that observed in mice with deletion of the entire *Pcdh*γ cluster (Chen et al., 2012; Carriere et al., 2020; Mancia Leon et al., 2020). Moreover, deletion of only *Pcdhγc4* fully recapitulates interneuron apoptosis caused by deletion of the entire *Pcdh*γ cluster, suggesting that *Pcdhγc4* is the only crucial isoform required for neuronal survival (Figure 3E) (Garrett et al., 2019). Although genetic evidence suggested that *Pcdhγc4* contributes to interneuron survival, analyses of double or tri-cluster deletion mice revealed that *Pcdh*α and β clusters also have cooperative roles in neuronal survival. For example, cell apoptosis in the spinal cord of the *Pcdh*βγ double-cluster or tri-cluster deletion mice is much more severe than that of the *Pcdh*γ-cluster deletion mice, although deletion of *Pcdh*α or *Pcdh*β themselves reveals no apoptosis (Hasegawa et al., 2016). In addition, double deletion of *Pcdh*α and *Pcdh*γ also leads to more severe apoptosis of retina cells than the *Pcdh*γ deletion alone (Ing-Esteves et al., 2018). It’s possible that members of Pcdhβ and Pcdhγ contribute to neuronal survival by acting as carrier isoforms to facilitate Pcdhγc4 to be delivered to the cell surface.

### A Role of Pcdhα in Myelination

Pcdhα has been observed to be strongly expressed in the developing, but not mature, axons at the late embryonic and early postnatal stages in mice. Specifically, Pcdhα expression is negatively correlated with the increasing expression levels of myelination markers during development (Morishita et al., 2004). In addition, deletion of the *Pcdh*α cluster in mice results in delayed oligodendrocyte maturation and defects in myelination (Yu et al., 2012). Moreover, imaging by transmission electron microscopy revealed the reduced ratio of myelinated nerve fibers and abnormal myelin sheaths in the *Pcdh*α deletion mice (Lu et al., 2018). Consistently, patients with a deletion in the 5q31.3 region, which covers the *Pcdh*α cluster, show delayed myelination (Shimojima et al., 2011). Further investigation of how cPcdhs participate in myelination processes is urgently needed.

### Clustered Pcdhs Regulate Spine Morphogenesis and Synaptogenesis

The establishment of proper synaptic connections in the brain is central to information processing. Subcellular fractionation and microscopic imaging studies have shown that isoforms of Pcdhα and Pcdhγ are located at synaptic junctions (Kohmura et al., 1998; Wang et al., 2002b; Phillips et al., 2003; Lefebvre et al., 2008; Garrett and Weiner, 2009). In addition, two isoforms of Pcdhβ, β16 and β22, have been found to be enriched in subsets of synapses in the retina and cerebellum (Junghans et al., 2008; Puller and Haverkamp, 2011; Nuhn and Fuerst, 2014). Finally, isoforms of Pcdhγ are found to accumulate at axodendritic and dendrodendritic synapses (Fernandez-Monreal et al., 2009). These expression patterns suggest a role of cPcdhs in synaptogenesis.

Deletion of *Pcdh*γ leads to a significant loss of synapses in the mouse spinal cord (Wang et al., 2002b). To specifically investigate the role of cPcdhs in synapse development, apoptosis is blocked by BAX mutation in the *Pcdh*γ deletion mice. In this case, the spinal cord still displays decreased synaptic density, suggesting a direct role of Pcdhγ in synaptogenesis (Weiner et al., 2005). In contrast, blocking apoptosis in *Pcdh*γ-deficient retina by mutating BAX reveals normal synaptic numbers (Lefebvre et al., 2008). In addition, the decreased VGAT+ and increased VGLUT1+ synapses seen in the *Pcdh*γ-deficient spinal cord are secondary to interneuron apoptosis (Chen et al., 2012). Another study showed that synapse development requires Pcdhγ-mediated contacts between astrocytes and neurons (Garrett and Weiner, 2009). Specifically, deletion of *Pcdh*γ in astrocytes *in vivo* reduces both excitatory and inhibitory synapses in a contact-dependent mechanism (Garrett and Weiner, 2009). Finally, reciprocal synaptic connections between sister neurons derived from the same neural stem cell are impaired by deletion of the three *Pcdh* clusters (Tarusawa et al., 2016).

In the cortical neurons of *Pcdh*γ knockout mice, only thin spines are slightly increased (Molumby et al., 2017). In addition, overexpression of a single *Pcdh*γ gene, *γa1*, significantly decreases spine density through repressing the postsynaptic cell adhesion molecule neuroligin-1 (Molumby et al., 2017). However, conditional knockout of *Pcdh*γ in the olfactory granule cells decreases dendritic spines (Ledderose et al., 2013). In addition, in *Pcdh*α deletion mice, a significant decrease of spine density was observed in hippocampal Golgi staining *in vivo* and cultured hippocampal neurons *in vitro* (Suo et al., 2012). Similar phenotype was found in *Pcdh*γ knockdown hippocampal neurons (Suo et al., 2012). In summary, cPcdhs are implicated in various aspects of synapse development in a cell-type dependent manner.

## A Signaling Pathway Linking Clustered Pcdhs to Lamellipodial Formation and Actin Cytoskeletal Dynamics

A growing body of evidence supports the vital roles of cPcdhs in neuronal survival and migration, dendritic arborization and self-avoidance, spine morphogenesis and maturation, axonal targeting and tiling, as well as synaptogenesis (Figure 2) (Wang et al., 2002b; Weiner et al., 2005; Chen et al., 2012, 2017; Garrett et al., 2012; Lefebvre et al., 2012; Suo et al., 2012; Katori et al., 2017; Mountoufaris et al., 2017; Fan et al., 2018; Ing-Esteves et al., 2018; reviewed in Mountoufaris et al., 2018). These diverse functions of cPcdhs are all subserved by the intracellular signaling pathway via their conserved CDs. Interestingly, Pcdhα cluster contains a WIRS (Wiskott-Aldrich syndrome family verprolin homologous protein (WAVE) interacting receptor sequence) motif in CD which can directly bind WRC (WAVE regulatory complex), linking to actin filament branching via the Arp2/3 complex, to regulate lamellipodial dynamics and cytoskeletal remodeling (Fan et al., 2018).

Pcdhα also indirectly modulates actin dynamics through inhibiting Pyk2, which is a synaptic non-receptor tyrosine kinase. Pyk2, a known risk factor in neuropsychiatric diseases (Lambert et al., 2013), then regulates conformational changes of WRC via Rac1 (Fan et al., 2018). The Pcdh β and γ proteins, on the other hand, do not contain the WIRS motif. However, they may also modulate actin dynamics through forming *cis*-heterodimers with Pcdhα (Thu et al., 2014; Rubinstein et al., 2015; Goodman et al., 2017), or through the shared downstream effector Pyk2 (Chen et al., 2009; Suo et al., 2012) (reviewed in Wu and Jia, 2020). Thus, this actin cytoskeletal dynamic regulation via Pyk2 and WRC is a general underlying mechanism for various functions of cPcdhs (Fan et al., 2018). It is known that dysregulation of actin dynamics and synaptic structural plasticity is closely related to neuropsychiatric diseases (Forrest et al., 2018). In the following four sections, we discuss the genetic, epigenetic, 3D genomic, and environmental dysregulations of cPcdhs in neuropsychiatric diseases.

## Genetic Variants of Clustered Pcdhs in Mental Disorders

Patients with the 5q31.3 deletion, which includes the three *Pcdh* clusters, show severe neurodevelopmental delay, encephalopathy associated with myelination defects, and hypotonia (Table 1) (Shimojima et al., 2011; Brown et al., 2013). In addition, human genetic studies revealed that deletion of a 5q31.3 fragment covering the *Pcdh*α cluster is associated with poor music perception, suggesting its role in human higher cognitive functions (Table 1) (Ukkola-Vuoti et al., 2013).

**TABLE 1:** Genetic/epigenetic dysregulation of the three *Pcdh* clusters causes various brain disorders.

Disorder	Gene	Alteration	References
Aging	Pcdh clusters	Differential methylation	Rakyan et al., 2010; Bell et al., 2012; Salpea et al., 2012; Hannum et al., 2013; McClay et al., 2014; Slieker et al., 2016; Kim et al., 2018a
Alzheimer’s disease	Pcdhγc5	Altered expression	Li et al., 2012 (rat) Li et al., 2017 (mouse)
	Pcdh clusters	Altered expression	Nativio et al., 2018
			Meyer et al., 2019
Antipsychotic medication	Pcdh clusters	Differential methylation	Melka et al., 2014; Nakazawa et al., 2017
Autism	Pcdhα cluster	SNP	Anitha et al., 2013
	Pcdh clusters	*De novo* mutations	Iossifov et al., 2014
	Pcdh clusters	SNV, CNV	Krumm et al., 2015
	Pcdhα12	Differential methylation	Stathopoulos et al., 2020
Bipolar disorder	Pcdh clusters	Association	Hong et al., 2004; Herzberg et al., 2006
	Pcdhα cluster	SNP	Pedrosa et al., 2008
Child maltreatment	Pcdh clusters	Differential methylation	McGowan et al., 2011 (rat) Suderman et al., 2012 (rat and human)
Developmental delay	Pcdh clusters	5q31.3 microdeletion	Shimojima et al., 2011; Brown et al., 2013
Down syndrome	Pcdhγa2	Differential methylation	Mendioroz et al., 2015
	Pcdhγ cluster	Differential methylation	El Hajj et al., 2016; Almenar-Queralt et al., 2019
	Pcdhγ cluster	Altered expression	Ponroy Bally et al., 2020
			Gonzales et al., 2018
Dyslexia	Pcdhγ cluster	SNP	Naskar et al., 2018
Extreme obesity	Pcdhγ cluster	Deletion	Su et al., 2010 (mouse)
	Pcdh clusters	Rare variants	Mariman et al., 2014, 2015; Moon et al., 2017
Fetal alcohol spectrum disorder	Pcdh clusters	Differential methylation	Laufer et al., 2015, 2017
Poor music perception	Pcdhα cluster	5q31.3 microdeletion	Ukkola-Vuoti et al., 2013
Major depressive disorder	Pcdhγ cluster	Altered expression	Garafola and Henn, 2014 (rat)
	Pcdhαc2	Deletion	Chen et al., 2017 (mouse)
	Pcdhα6, α8	Altered expression	Vadodaria et al., 2019
	Pcdhα7, α8	Altered expression	Hall et al., 2020
Schizophrenia	Pcdh clusters	Association	Schwab et al., 1997; Straub et al., 1997; Schizophrenia Working Group of the Psychiatric Genomics Consortium, 2014; Pardinas et al., 2018; Walker et al., 2019
	Pcdh clusters	3D genome	Rajarajan et al., 2018
	Pcdh clusters	Altered Pcdh pathway	Shao et al., 2019
	Pcdhα7, α8	Altered expression	Hall et al., 2020

### Clustered *Pcdh* Genes in Autism Spectrum Disorders (ASD)

Autism spectrum disorders (ASD) are common neurodevelopment diseases with social, communicational, and behavioral impairments (Betancur et al., 2009). Genetic studies have identified *cPcdhs* as candidate genes for ASD. First, whole exome sequencing identified *de novo* mutation of Pcdhβ4 D555H in sporadic autism probands (O’Roak et al., 2012). Second, five single-nucleotide polymorphisms (SNPs) of *Pcdh*α are reported to have significant genetic associations with autism in a large cohort of 841 families (Anitha et al., 2013). Third, whole exome sequencing revealed that single nucleotide variants (SNVs) within the *Pcdh* clusters are associated with ASD (Iossifov et al., 2014; Krumm et al., 2015). Finally, abnormal CpG methylation patterns of *Pcdhα12* are found in a South African ASD cohort (Stathopoulos et al., 2020).

### Clustered *Pcdh* Genes in Bipolar Disorders

The 5q31 region is found to be associated with bipolar disorders (Hong et al., 2004; Herzberg et al., 2006). Specifically, patients with bipolar disorders showed a striking increase in homozygosity of a minor allele of the *Pcdh*α cluster, in which a SNP within the *HS5-1* enhancer is found to be associated with aberrant *Pcdh*α expression (Pedrosa et al., 2008).

### Clustered *Pcdh* Genes in Dyslexia

A set of SNPs within the *Pcdh*γ cluster is dominantly inherited in a family with dyslexia, a neurodevelopmental disorder characterized by reading and writing difficulties (Naskar et al., 2018). Two of them alter amino acids within EC2 and EC3 domains of Pcdh γa3 and γa4, respectively (Naskar et al., 2018). These missense SNPs located in the two critical ectodomains required for homophilic interactions, which might alter cell recognition and contribute to aberrant neuronal circuits of dyslexia.

### Clustered *Pcdh* Genes in Extreme Obesity with Behavioral Abnormality

In patients with extreme obesity, *cPcdhs* have a significantly higher frequency of rare variants than in general population, indicating an association between rare variants of the *Pcdh* clusters and extreme obesity (Table 1) (Mariman et al., 2014, 2015). In addition, copy number variations (CNVs) of two *Pcdh* genes, *Pcdhβ7* and *Pcdhβ8*, are associated with increased body mass index (Moon et al., 2017). In mice, *Pcdh*γ regulates hypothalamic feeding circuitry (Su et al., 2010), which is known to be involved in extreme obesity. In particular, conditional knockout of *Pcdh*γ in mouse hypothalamic neurons causes excessive feeding behaviors and defects in energy homeostasis, leading to extreme obesity (Su et al., 2010). These studies suggest that *cPcdhs* may play an important role in regulating neural circuits controlling feeding behavior, resulting in extreme obesity.

## Epigenetic Dysregulations of Clustered Pcdhs in Mental Disorders

### Clustered *Pcdhs* in Down Syndrome

Down syndrome (DS) is a genetic disorder with intellectual disabilities caused by an extra copy of chromosome 21. In the developing DS brain tissues, 5′ alternate isoforms of the *Pcdh*γ cluster are significantly hypermethylated (Table 1) (Mendioroz et al., 2015; El Hajj et al., 2016). In addition, in postmortem DS brains, 3′ alternate isoforms of the *Pcdh*γ cluster retain the fetal-like methylation state (Almenar-Queralt et al., 2019). Consequently, expression levels of these isoforms remain high in adult brain tissues, similar to that of the fetal stage, supporting a model of delayed brain maturation in the DS patients (Almenar-Queralt et al., 2019). Moreover, human DS iPSC-derived astrocytes show a transcriptional profile between fetal and mature stages, further supporting the delayed maturation model of DS (Ponroy Bally et al., 2020). Furthermore, these DS astrocytes show reduced levels of the *Pcdh*γ expression and have selective impairments in Pcdhγ-mediated cell adhesion (Ponroy Bally et al., 2020). Finally, nearly all of the *cPcdh* genes are downregulated in trisomic iPSCs comparing to the genetically edited disomic iPSCs derived from the same DS patient (Gonzales et al., 2018). Considering the important functions of cPcdhs in brain development, it’s possible that dysregulated cPcdhs may be related to defective synaptogenesis and neurite growth seen in DS brains.

### Clustered *Pcdhs* in Alzheimer’s Disease (AD)

In humans, the expression of *cPcdh* genes is frequently found to be altered in AD. For example, *Pcdhαc2* and *Pcdhγc5* are decreased while subsets of alternate *Pcdh*γ isoforms are increased in the lateral temporal lobe of postmortem AD brains (Nativio et al., 2018). Interestingly, APOE4, a prominent genetic risk allele for late-onset AD, causes *cPcdh* gene upregulation in iPSC-derived cerebral organoids (Meyer et al., 2019).

In mice, loss of excitatory or inhibitory synapses has been implicated in the pathogenesis of AD. For example, Pcdhγc5 specifically interacts with GABA receptors through its cytoplasmic domain to stabilize and promote inhibitory GABAergic synaptic transmission (Li et al., 2012). The expression levels of Pcdhγc5 in GABAergic synapses are increased in hyperexcitatory conditions. The increased Pcdhγc5 enhances synaptic inhibition and elevates GABAergic protein levels, which may contribute to pathogenic excitatory/inhibitory imbalance in an AD mouse model (Table 1) (Li et al., 2017). As a downstream effector of cPcdhs, the cell-adhesion kinase Pyk2 has been identified as a major susceptibility gene for late-onset AD (Lambert et al., 2013). Consistently, overexpression of Pyk2 results in synapse loss and learning deficit (Lee et al., 2019; Salazar et al., 2019). Therefore, dysregulation of *cPcdhs* or disruption of its intracellular signaling pathway may contribute to AD pathogenesis.

### Clustered *Pcdhs* in Major Depressive Disorders (MDD)

Reduced serotonin levels have been implicated in the pathogenesis of MDD and selective serotonin reuptake inhibitors (SSRIs) are the most commonly used antidepressants (Belmaker and Agam, 2008). In humans, altered expression patterns of members of the *Pcdh*α cluster are detected in serotonergic neurons derived from patients of SSRI-resistant MDD (Vadodaria et al., 2019). In addition, low expression levels of *Pcdhα7* and *Pcdhα8* in the prenatal brain are associated with MDD (Hall et al., 2020). Moreover, in rats with learned helplessness, a model of depression, members of the *Pcdh*γ cluster are upregulated in CA1 neurons (Garafola and Henn, 2014). Finally, in mice *Pcdhαc2* is specifically expressed in serotonergic neurons and knockout of the *Pcdh*α cluster leads to depressive-like behaviors (Chen et al., 2017). In conjunction with the important role of cPcdhs in serotonergic axon wiring (Chen et al., 2017), these findings suggest that uneven distribution of serotonergic fibers by cPcdh dysregulation could lead to reduced serotonin signaling in various brain regions.

### Clustered *Pcdhs* in Schizophrenia

Clustered *Pcdhs* have been identified as susceptible loci for schizophrenia (Schwab et al., 1997; Straub et al., 1997; Pardinas et al., 2018; Walker et al., 2019). In particular, large-scale genome-wide association studies have revealed that the *cPcdh* locus is associated with schizophrenia (Schizophrenia Working Group of the Psychiatric Genomics Consortium, 2014). In addition, high expression levels of members of the *Pcdh*α cluster, *Pcdhα7* and *Pcdhα8*, are associated with schizophrenia (Hall et al., 2020). Finally, cPcdhs and their downstream effector PKC are dysregulated in hiPSC-derived cortical interneurons from schizophrenic patients (Shao et al., 2019).

Epigenetic modifications, especially DNA methylation of gene promoters, play a major role in *cPcdh* regulation (reviewed in Wu and Jia, 2020). Antisense transcription of long non-coding RNAs leads to demethylation of *cPcdh* promoters (Canzio et al., 2019). CTCF then binds the unmethylated *cPcdh* promoters and brings them in close contacts with remote enhancers via long-distance chromatin interactions to activate *cPcdh* transcription (Guo et al., 2012). Collectively, these studies suggest that epigenetic dysregulation of *cPcdhs* is related to various mental disorders. Since epigenetic and higher-order chromatin regulations are a general phenomenon, it is very likely that these regulations also affect other cell-surface neural receptors.

## 3D Genome Dysregulations of Clustered Pcdhs in Mental Disorders

**FIGURE 4:**
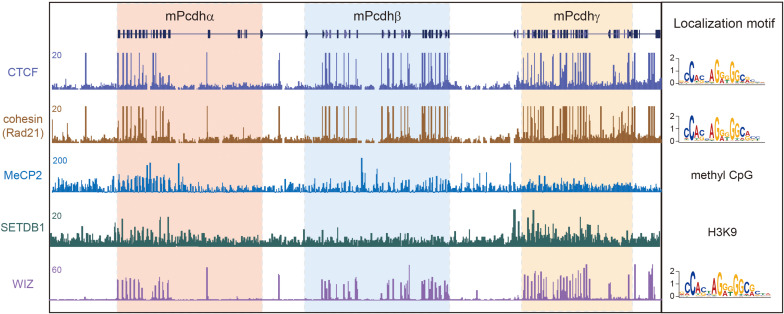
3D genome architectural proteins regulate *cPcdh* gene expression. Shown are schematic binding patterns of architectural proteins in the three *Pcdh* clusters.

The proper expression of the *cPcdh* genes requires specific long-range enhancer-promoter contacts in the 3D nuclear space. Mutations in genes encoding 3D genome architectural proteins, such as CTCF, cohesin, MeCP2, SETDB1, and WIZ, dysregulate *cPcdh* genes through altering higher-order chromatin contacts (Figure 4). Therefore, it’s interesting to note that an increasing list of mutations in genes encoding 3D genome architectural proteins is linked to brain disorders. We suggest that many complex neuropsychiatric diseases of mutations of 3D genome regulators are ‘actually’ resulted from cPcdh dysregulations.

### 3D Genome Dysregulation of Clustered *Pcdhs* by CTCF

*De novo* mutations of CTCF in humans causes the type 21 of autosomal dominant mental retardation (MRD21) (Gregor et al., 2013; Bastaki et al., 2017; Hori et al., 2017; Chen et al., 2019; Konrad et al., 2019). This rare condition is first described in four individuals with intellectual disability, microcephaly, and growth retardation. Genetic studies revealed one individual with CTCF missense mutation, two with CTCF frameshift mutations, and one with CTCF deletion (Gregor et al., 2013). A subsequent study reported an additional individual of MRD21 with a different *de novo* CTCF frameshift mutation (Bastaki et al., 2017). Recent studies independently reported 2, 3, and 39 additional individuals of MRD21 with pathogenic variants of CTCF (Hori et al., 2017; Chen et al., 2019; Konrad et al., 2019). Finally, a human genetic study showed strong associations between CTCF SNPs and schizophrenia in multiple cohorts (Juraeva et al., 2014). Given that CTCF mutations or variants dysregulate *cPcdh* genes, it’s likely that this *cPcdh* dysregulation is responsible for complex brain disorders caused by CTCF mutations or variants.

In mice, conditional knockout of CTCF in cortical neurons downregulates nearly all of the *cPcdh* genes. These mice display abnormal behavior and growth retardation (Hirayama et al., 2012). Strikingly, every member of the *cPcdh* genes carrying CBS elements is downregulated, suggesting that long-distance chromatin looping between enhancers and promoters is impaired. Histological analyses revealed the loss of the functional somatosensory circuits and the reduction of dendritic arborizations in CTCF knockout mice, reminiscent of the phenotypes of *cPcdh* deletion (Hirayama et al., 2012). In addition, conditional CTCF knockout in excitatory or inhibitory neurons impairs long term memory and cortical synaptic plasticity (Kim et al., 2018b). Finally, RNA-seq experiments revealed a predominant dysregulation of the *cPcdh* genes (Kim et al., 2018b). In conjunction with the known role of CTCF in the *cPcdh* 3D regulation, it is possible that neuropsychiatric symptoms in human CTCF mutations are related to the dysregulation of *cPcdh* genes.

### 3D Genome Dysregulation of Clustered *Pcdhs* by Cohesin and its Regulators

The cohesin complex, which mediates chromatin loop extrusion and sister chromatid cohesion, is central for 3D genome folding. Mutations of cohesin or of its regulators have been shown to be associated with a large set of complex human diseases, collectively called cohesinopathies (Liu and Krantz, 2008). The symptoms of cohesinopathies vary in different mutations but share the same manifestations such as cognitive retardation and intellectual disability. Cornelia de Lange syndrome (CdLS), the best characterized cohesinopathy, is caused by mutations of cohesin regulators, such as NIPBL and HDAC8, or of cohesin subunits, including SMC1A, SMC3, and RAD21 (Bose and Gerton, 2010). Roberts syndrome, which is phenotypically related to CdLS, is caused by mutations of the cohesin regulator ESCO2 (Liu and Krantz, 2008). Similar to CTCF, knockdown of cohesin in cell lines significantly downregulates the expression of *cPcdh* genes (Guo et al., 2012). Interestingly, only the SA1 but not SA2 subunit of cohesin is located at *cPcdh* promoters and regulates their activities (Remeseiro et al., 2012). Collectively, cognitive impairments in cohesinopathies with mutations of cohesin subunits or of its regulators may result from their 3D genome dysregulations of the *cPcdh* genes.

### 3D Genome Dysregulation of Clustered *Pcdhs* by MeCP2

The Rett syndrome is a rare, severe type of ASDs caused by mutations in the X-linked *MeCP2* (methyl-CpG binding protein 2) gene (Amir et al., 1999). In addition, *Pcdhβ1* is upregulated in both the *MeCP2* mutant mice and postmortem brains of Rett syndrome patients (Miyake et al., 2011). Moreover, in *MeCP2* mutant mice, several members of the *Pcdh* clusters are dysregulated (Chahrour et al., 2008). Finally, in the human *cPcdh* locus, each of the 15 *Pcdh*α, 16 *Pcdh*β, and 22 *Pcdh*γ genes is associated with a CpG island (Wu et al., 2001) and methylation of a CpG dinucleotide within the *cPcdh* promoter CTCF sites precludes CTCF binding (Guo et al., 2012). Given the known role of MeCP2 in gene regulation via the binding to methylated CpG islands, MeCP2 and CTCF may bind mutual exclusively to the *cPcdh* promoters. Thus, the dysregulation of cPcdhs by MeCP2 mutations may result from altering 3D genome configuration via CTCF.

### 3D Genome Dysregulation of Clustered *Pcdhs* by SETDB1

Deletion of *SETDB1* (SET domain bifurcated 1), which encodes a histone H3K9 methyltransferase, results in a five-hundred-fold increase of *cPcdh* transcripts in the mouse cortex compared to the rest of the genome (Jiang et al., 2017). SETDB1 protects the *cPcdh* locus from excessive CTCF binding to maintain the *cPcdh* superTAD structure (Jiang et al., 2017). Interestingly, the 5’ boundary of the *cPcdh* superTAD, which enriches with H3K9me3 histone modifications (Figure 4), contains a haplotype significantly associated with schizophrenia (Schizophrenia Working Group of the Psychiatric Genomics Consortium, 2014; Jiang et al., 2017). This region regulates the expression of *cPcdh* genes through long-range chromatin loops across hundreds of kilobases (Schizophrenia Working Group of the Psychiatric Genomics Consortium, 2014; Rajarajan et al., 2018). Within this region a schizophrenia-risk SNP (rs111896713) significantly affects the expression of *Pcdh*α genes in a dosage-dependent manner (Fromer et al., 2016; Rajarajan et al., 2018).

Mutations of SETDB1 itself are also associated with psychiatric disorders such as autism (Cukier et al., 2012). In addition, the expression of SETDB1 is markedly increased in patients with Huntington’s disease (HD) (Ryu et al., 2006). In the mouse model of HD, 37 out of 58 *cPcdh* genes are striking dysregulated as the size of huntingtin CAG repeats increases (Langfelder et al., 2016). Together, cognitive defects associated with SETDB1 mutations may result from the dysregulation of *cPcdhs*.

### 3D Genome Dysregulation of Clustered *Pcdhs* by WIZ

WIZ (Widely interspaced zinc finger-containing protein) forms a heteromeric H3K9 methyltransferase complex with G9a and GLP. In autism patients, exon resequencing identified non-synonymous variants in WIZ and its partners G9a/GLP (Balan et al., 2014). Haploinsufficiency of GLP causes the Kleefstra syndrome, a multi-system syndrome associated with intellectual disability, neurodevelopmental delay, and neuropsychiatric diseases (Willemsen et al., 2012). WIZ is a modulator of chromatin loops and its genomic binding sites overlap with CTCF (Figure 4) (Isbel et al., 2016; Justice et al., 2020). Remarkably, only members of the *Pcdh*β cluster are dysregulated in WIZ mutant mice (Isbel et al., 2016). Interestingly, these mice display an anxiety-like phenotype (Isbel et al., 2016), consistent with the requirement for cPcdhs in neuronal development and connectivity. All in all, these human and mouse genetic studies suggest that mutations of genome architectural proteins may be involved in pathogenesis of complex neural diseases through dysregulation of *cPcdh* genes (Figure 4).

## Dysregulations of Clustered Pcdhs in Mental Disorders by Environmental Factors

### Clustered *Pcdhs* and Child Maltreatment

Early life experiences are known to have long-term effects on mental health and behavior through epigenetic reprogramming, including alterations in stress responses. *cPcdhs* display differential methylations in response to maternal cares. In children suffered from maltreatment or abuse, increased methylation levels are seen in promoters across the three *Pcdh* clusters, especially in *Pcdh*α (Table 1) (Suderman et al., 2012). In rat pups who received less licking and grooming from mothers, *cPcdh* promoters are hypermethylated and their expression levels are downregulated (McGowan et al., 2011; Suderman et al., 2012). These studies suggest that early life experience affects *cPcdh* expression through epigenetic modification of their promoters, which may contribute to the psychological abnormalities in abused children.

### Clustered *Pcdhs* and Fetal Alcohol Spectrum Disorders (FASD)

Similar to early life experiences, prenatal alcohol exposure during pregnancy, which can cause lifelong alterations in cognition and behavior, also influences the methylation states of the three *Pcdh* clusters (Laufer et al., 2015, 2017). In mouse models of FASDs and in children suffered from prenatal alcohol exposure, there are similar methylation changes in the *cPcdh* genes (Laufer et al., 2015, 2017).

### Clustered *Pcdhs* and Antipsychotic Medication

Olanzapine, an antipsychotic medication commonly used for treating bipolar disorders and schizophrenia, causes methylation changes in the cortex in a rat model. Interestingly, the promoter regions of *cPcdhs* showed prominent methylation alterations in the cerebrum and hippocampus (Table 1) (Melka et al., 2014). In addition, clozapine, the most effective medication for treatment-resistant schizophrenia, causes differential expression of *cPcdh* genes in iPSC-derived neurons (Nakazawa et al., 2017). Thus, antipsychotic drugs may exert their therapeutic effects through altering the regulation of *cPcdhs*.

### Clustered *Pcdhs* and Brain Aging

Epigenetic alteration is a hallmark of aging. Interestingly, many age-related differentially methylated regions are found to be located within the three *Pcdh* clusters (Rakyan et al., 2010; Bell et al., 2012; Hannum et al., 2013; Slieker et al., 2016). In addition, *Pcdh*α is prominently differentially methylated during aging (McClay et al., 2014). Moreover, epigenetic modifications in the three *Pcdh* clusters are reprogrammed during aging (Salpea et al., 2012). Finally, a 134-twin aging study revealed that methylation states of *cPcdh* promoters correlate with biological ages (Kim et al., 2018a). Altogether, various environmental factors can influence methylation states of the *Pcdh* clusters, leading to their abnormal expression patterns, which may be related to mental disorders and behavioral abnormalities.

## Concluding Remarks

Clustered Pcdhs are thought to function as molecular identity codes for individual neurons to discriminate self from non-self (Lefebvre et al., 2012; Mountoufaris et al., 2018; Honig and Shapiro, 2020; Sanes and Zipursky, 2020; Wu and Jia, 2020). The tremendous diversity afforded by unique variable and constant genomic organization (Figure 1), the stochastic promoter choice realized by topological chromatin looping and balanced enhancer contacts (Figure 1A), and amazing structural recognition evolved through promiscuous *cis*-interactions and stringent homophilic *trans*-interactions (Figure 2) enable billions of neurons to achieve neurite self-avoidance and to establish proper connectivity in the brain. A key unresolved question is how cPcdh homophilic adhesions are transformed into neurite repulsion. This is likely achieved through common intracellular signaling transduction pathways with Pyk2, FAK, Ret, and WRC complex, eventually leading to actin dynamics and cytoskeletal remodeling (Figure 2). Shedding the extracellular domain by metalloproteinase may be essential for separating the homophilic-interaction-bridged plasma membranes. Finally, genetic studies provide strong evidence that *cPcdh* genes are central for neurodevelopment and neurite morphogenesis in the brain (Figure 3).

The diverse roles of *cPcdh* genes in neural circuit formation suggest that their mutations or dysregulations may be involved in complex brain disorders. Indeed, numerous studies reported mutations of *cPcdh* genes in Alzheimer’s disease, ASDs, bipolar disorders, major depressive disorders, schizophrenia, behavioral abnormalities, and several neurodevelopmental disorders with cognitive impairments (Table 1). In addition, mutations of 3D genome architectural proteins or regulators of *cPcdh* genes such as CTCF, cohesin, MeCP2, SETDB1, and WIZ cause a wide variety of brain disorders (Figure 4). These overwhelming data suggest that mutations or dysregulations of *cPcdh* genes play an important role in the pathogenesis of brain disorders.

In summary, growing lines of evidence suggest the important roles of *cPcdh* genes in complex mental or brain disorders. However, most brain and mind diseases have complex etiology and pathogenesis. How these diseases are initiated and progressed through genetic mutations and epigenetic dysregulations of *cPcdh* genes remain largely unknown. A deeper understanding of regulatory mechanisms and biological functions of *cPcdh* genes may be the key to understand the etiology and pathogenesis and to facilitate developing rational therapeutic strategies in the future.

## Author Contributions

Both authors listed have made a substantial, direct, and intellectual contribution to the work and approved it for publication.

## Conflict of Interest

The authors declare that the research was conducted in the absence of any commercial or financial relationships that could be construed as a potential conflict of interest.
